# Temporal and geographic heterogeneity of the association between socioeconomic position and hospitalisation in Italy: an income based indicator

**DOI:** 10.1186/1475-9276-8-33

**Published:** 2009-09-17

**Authors:** Patrizia Schifano, Chiara Marinacci, Giulia Cesaroni, Valeria Belleudi, Nicola Caranci, Antonio Russo, Carlo A Perucci

**Affiliations:** 1 Department of Epidemiology, Health Local Unit ASL RME, Rome, Italy; 2 Epidemiology Unit, Piedmont Region, Italy; 3 Regional Health Care Agency of Emilia Romagna, Italy; 4 Epidemiology Unit, Local Health Authority of Milan, Italy

## Abstract

**Background:**

The inverse association between socioeconomic position (SEP) and health has been extensively explored in Italy; however few studies have been carried out on the relationship between income inequalities and health status or health services utilisation, particularly at a local level.

The objective of this study is to test the association between the demand for hospital care and a small area indicator based on income in four Italian cities, over a four-year period (1997-2000), in the adult population.

**Methods:**

Census Block (median 260 residents) Median per capita Income (CBMI) was computed through record linkage between 1998 national tax and local population registries in the cities of Rome, Turin, Milan and Bologna (total population approximately 5.5 million). CBMI was linked to acute hospital discharges among residents, based on patient's residence.

Age-standardized gender-specific hospitalisation rates were computed by CBMI quintiles (first quintile indicating lowest income), overall, and by city and year. Heterogeneity of the association between income level and hospitalisation was analysed through a Poisson model.

**Results:**

We found an inverse association between small area income level and hospitalisation rates, which decreased continuously from 153 per 1000 inhabitants in the first quintile to 107 per 1000 inhabitants in the fifth quintile. Income differences in hospitalisation were confirmed in each city and year. However, the magnitude of the association and the absolute level of hospitalisation rates were quite different in each city and tended to slightly decrease over time in all cities considered, except Bologna.

**Conclusion:**

Our study confirms an inverse association between income level and the use of hospitalization in four Italian cities, using a small area economic indicator, based on population tax data. Further analysis of the association between income and cause-specific hospitalization rates will allow to better understand the capability of the Italian National Health System to compel with socio-economic inequalities in health needs.

Furthermore the SEP indicator we propose can represent a contribution to the improvement of tools for monitoring inequalities in health and in health services utilization.

## Introduction

The association between socioeconomic position (SEP) and health has been studied extensively both in Europe and in the United States [1-4] and it has been confirmed by studies using several SEP and health indicators and in different countries, including Italy [5-11]. Fewer data and inconsistent results are available on income inequalities in health services utilisation, varying according to the specific healthcare branch analysed, but also influenced by the specific systems and policies [12-14].

However a non-linear inverse relationship between health and income level has been proved, independent of the health outcomes analysed [15-18]; results from longitudinal studies [19] have shown that even though average lifetime income appears more significant in explaining differences in health, current income explains important components of health inequalities. Furthermore income level has been seen to be more important than income change in explaining health inequalities.

In order to optimally studying the relationship between SEP and health or health service utilisation, connection between health, health care and measures of SEP would be needed at the individual level; in Italy this is available, on a national scale, only in the National Health Interview Survey [20]. This survey collects a range of measures of self reported health or health service consumption and SEP on a representative sample of the population; however it is repeated every 5 years, and it does not include income information. An important body of Italian research aimed at analysing socioeconomic inequalities in health or health care is in fact locally based [11,21-24]. These studies are based on both individual and area socioeconomic indicators, mainly using decennial census information or ad hoc individually collected data [25], and their record linkage to health or health services utilization data. However census does not collect income information and composite SEP indices, developed from census data, are often context specific and not comparable.

To use official registries to collect income information would be the more appropriate and convenient way. Also, the use of area-level indicators as a proxy of individual information can be a way of addressing confidentiality issues on income data. Furthermore a relevant body of research shows that small area indicators are able to minimize the misclassification due to attributing to individuals an aggregated value in the analysis of socioeconomic inequalities in health [25-27].

The choice of the outcome measure is also an issue in this field of research. Hospitalization rates are among the most used outcome measures, mainly because of their wide availability and of their robustness. However they can be considered as a proxy both of the health status of a population as of their propensity to the use of health services. In our opinion the specific characteristic of the Italian national health service makes hospitalization rates more appropriate as a proxy of health utilization than health status. However both aspects are necessarily comprehended in this measure.

Our study is part of a National Project aimed at measuring equity in hospitalization (Ministry of Health - 2000 Research Program - "Special Programs" ex art. 12 c. 2 lett. B) D. Lgs. 502/92). The starting point of the project was to build an economic indicator to measure inequalities, applicable and comparable in all cities participating at the project.

This study presents the feasibility to develop a tax-based small area indicator of income, easily reproducible in different contexts, and tests its association with the demand for hospital care in four Italian cities, located over four different Italian regions, over a four year period (1997-2000).

## Methods

### Study Design

Cohort retrospective study, based on administrative data.

### Study Population

Individual demographic data of all city residents as of the 1^st ^of January of each year in the period 1997-2000 were extracted from the population register of each participating city: Rome (2.8 million inhabitants), Bologna (400,000 inhabitants), Turin (900,000 inhabitants) and Milan (1.4 million inhabitants).

### Hospital Admissions

All acute hospital discharges between the 1^st ^of January 1997 and the 31^st ^of December 2000, of residents in the study cities at the 1^st ^of January of each of the considered year, were selected from the Hospital discharge data Information System (HIS). We excluded subjects younger than 15 days old, rehabilitation admissions, day hospital admissions, and admissions of residents occurring outside the region of residence.

### Income Data

A median family equivalent income index was calculated for each census block (CB) in the participating cities. The mean number of inhabitants per CB varied between 500, for Rome, to 200 for Bologna.

**Figure 1:**
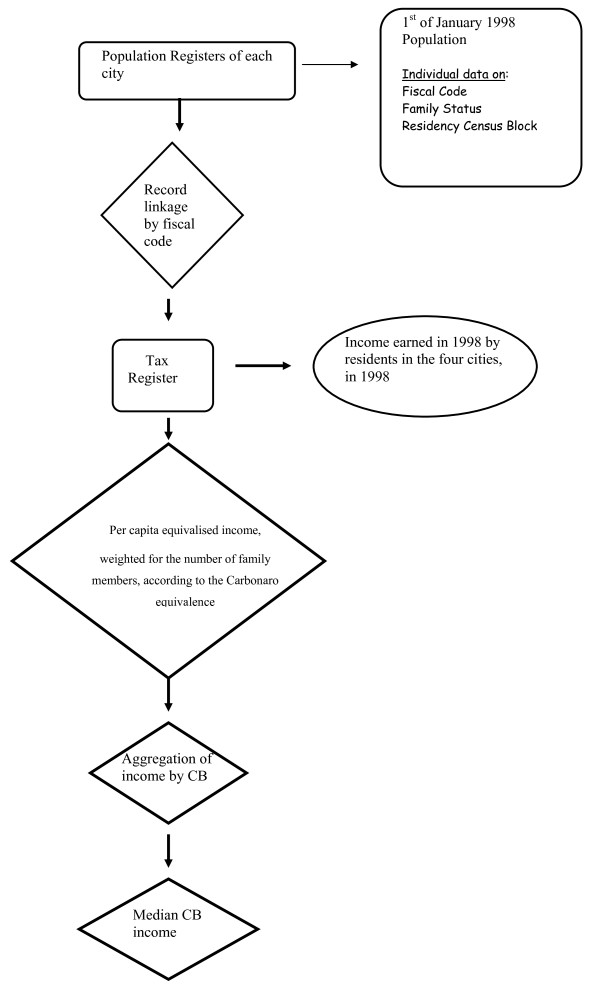
**Deciles and quintiles of the distribution of CBMI**. In order to obtain categorical values for the income indicator, for each city we calculated the deciles and quintiles of the distribution of CBMI.

Income earned in 1998 (tax returns of from 1999) were extracted from the Italian Tax Register for all residents in the participating cities. A record linkage between the Tax Register and the Population Registers of the four cities as of the 1^st ^of January 1998 connected family status information to each study subject income data. Using these data we calculated the per capita equivalised income, weighted for the number of family members, according to the Carbonaro equivalence scale [28]. Income has been expressed in euros. Income data were then aggregated at the CB level, and the census block median income (CBMI) was calculated. Disposable income was used. CBs with fewer than 25% of resident families with valid income data were excluded, affecting 1.5% of CTs in Rome, 0.5% in Bologna and 1.6% in Milan. In order to obtain categorical values for the income indicator, for each city we calculated the deciles and quintiles of the distribution of CBMI (Figure 1). For a more synthetic description and to reduce the effect of extreme values in the lowest and highest class we decided to use quintiles of the income distribution (Q1 first quintile, Q5 fifth quintile), instead of deciles, for the analysis of income association with hospitalisation rate. The income index, computed at the CB level, is meant to be a proxy of the relative position of each subject given the income distribution of his city of residence. The choice of relative income position instead of absolute income position is justified by the aim of using the same indicator in different Italian regions, where the same level of income correspond to a different purchasing power and level of wealth.

### Statistical Analysis

Age-standardized (using population of Italy for 1998) gender-specific hospitalisation rates per 1000 inhabitants were calculated, by quintiles of CBMI for each city and year, and for the total. The observed gradients were synthesized and compared between cities, through the ratio between the highest and the lowest CBMI quintiles.

Rate ratios were estimated using a Poisson regression model. The outcome variable was the hospitalisation rate, while the covariates were income level, year, city, gender and age. Interactions among gender, income level, year and city was included.

## Results

### The median income data in the four cities

The record-linkage procedure between Tax Registry and the database of resident populations attributed income data to 89.43% of resident families in Bologna, 85.63% in Turin, 79.56% in Milan, and 74.79% in Rome. The median income level for each census block was computed and attributed to the CB.

**Figure 2:**
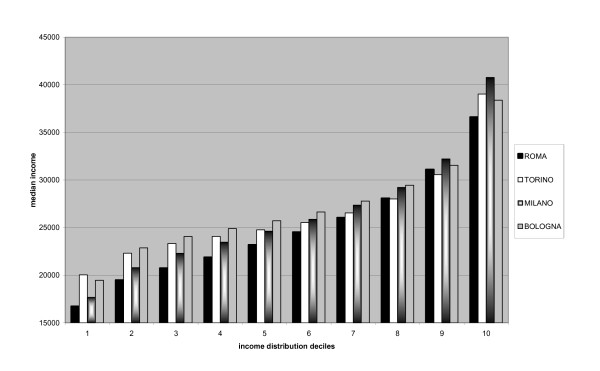
**The deciles of CBMI distribution for the four cities**. The deciles of CBMI distribution for the four cities.

The deciles of CBMI distribution for the four cities are shown in Figure 2.

For each decile, the median income value of that class is represented [29]. Rome and Milan showed the lowest income values in the lower deciles, while Milan and Turin had the highest income values for the higher income deciles. The tenth decile had a particular distribution among the cities; it represents a class with no upper limit, including uncommonly high income values.

### Declines in rates over time, by income levels, in absolute and relative terms

We analysed a total of 2,885,505 hospitalisations; data show an overall inverse relationship between income level and hospitalisation rates, that decreased continuously from 153.3 per 1000 inhabitants in the first quintile to 106.6 per 1000 inhabitants in the fifth quintile.

We stratified the analysis by gender, time and city, which confirmed income differences in hospitalisation in each of the strata considered. Table 1 reports hospitalization rates for each SEP level, and both the absolute and relative variation of rates ([(rate 2000 - rate 1997)/rate 1997] *100]) in the considered period, according to each level in each region. However, hospitalisation rates and the way in which they decrease over time are quite different in each city, and are generally higher for males than females, particularly in lower income levels (Table 1). Rome showed the highest values among the poorest; Bologna had the highest values across all the income levels; Turin had the lowest rates of hospitalisation for the richest and the poorest groups, while in Milan they were similar to Rome, even though lower among the poorest. In 1998, for example, the year to which income data refer, the highest hospitalisation rates in the first quintile were observed in Rome and Bologna both for males and females, while the lowest ones were observed in Turin; in the fifth quintile the highest rates were in Bologna, and the lowest in Turin, in both cases regardless of gender (Table 1). Turin always showed lowest hospitalisation rates, despite the income level; Bologna showed the highest levels of hospitalisation among both the poorest and the richest, with fewer differences between the extreme levels of income. Hospitalisations in Rome and Bologna were similar for the richest group.

**Table 1:** All cause hospital admissions age-standardized rates* (× 1000) and relative period increment, by sex and city. 1997-2000.

**Center**	**Gender**									
***Rome***	**Male**					**Female**				
	
	**Income index level**									
	
*year*	**Q1**	**Q2**	**Q3**	**Q4**	**Q5**	**Q1**	**Q2**	**Q3**	**Q4**	**Q5**
**1997**	175.3	149.1	137.6	128.2	111.2	168.7	147.3	135.4	125.8	111.7
**1998**	172.0	149.2	135.0	126.2	111.1	166.4	144.2	133.9	132.6	112.4
**1999**	166.9	139.8	129.7	122.5	107.4	158.4	138.0	127.2	126.6	107.5
**2000**	160.7	136.1	127.1	117.5	105.9	156.3	136.3	123.9	117.6	108.1
Absolute difference 1997-2000	14.58	13.00	10.50	10.70	5.37	12.43	11.00	11.50	8.20	3.65
Relative difference 1997-2000	0.08	0.09	0.08	0.08	0.05	0.07	0.07	0.08	0.07	0.03
*Bologna*										
*year*										
**1997**	177.1	161.3	160.1	146.6	135.6	175.7	158.8	145.7	144.4	138.1
**1998**	171.3	153.0	153.8	139.3	131.0	168.6	150.4	141.1	138.0	128.5
**1999**	161.7	147.5	140.8	138.6	126.0	160.9	146.5	139.3	126.9	128.1
**2000**	157.5	134.1	131.6	130.2	119.1	146.8	136.3	129.6	126.6	121.1
Absolute difference 1997-2000	19.59	27.20	28.50	16.40	16.51	28.88	22.50	16.10	17.80	16.99
Relative difference 1997-2000	0.11	0.17	0.18	0.11	0.12	0.16	0.14	0.11	0.12	0.12
*Milan*										
*year*										
**1997**	165.4	145.0	130.5	125.8	114.2	158.0	135.0	123.2	116.9	109.8
**1998**	165.4	141.9	131.9	124.6	113.3	155.1	132.9	125.3	115.4	110.5
**1999**	157.3	131.5	118.9	115.6	104.1	143.8	125.1	115.7	109.3	101.9
**2000**	142.4	120.8	110.5	110.0	99.1	130.1	113.4	105.5	101.2	100.0
Absolute difference 1997-2000	22.97	24.20	20.00	15.80	15.08	27.90	21.60	17.70	15.70	9.82
Relative difference 1997-2000	0.14	0.17	0.15	0.13	0.13	0.18	0.16	0.14	0.13	0.09
*Turin*										
*year*										
**1997**	146.6	131.4	122.5	117.5	97.4	141.8	129.0	121.4	114.8	95.1
**1998**	143.8	130.7	120.6	115.3	97.4	142.2	128.0	120.1	113.3	97.3
**1999**	140.4	129.8	121.0	113.0	95.1	138.2	129.4	123.6	115.0	98.1
**2000**	132.5	118.2	112.3	104.7	91.7	136.3	122.4	120.4	108.3	94.5
Absolute difference 1997-2000	14.16	13.20	10.20	12.80	5.65	5.57	6.60	1.00	6.50	0.67
Relative difference 1997-2000	0.10	0.10	0.08	0.11	0.06	0.04	0.05	0.01	0.06	0.01

*italian population, year 1998

The greatest reduction in hospitalisation rates, relative to the initial level of hospitalisation, was found in females from the lowest income class in Milan (-17,7% of hospitalisation per 1000 residents between 1997 and 2000). Bologna and Milan had the greatest drop in hospitalisation rates, but while in Bologna the decrease was quite uniform across rich and poor, male and female, (about -13% between 1997 and 2000), in Milan it was more than two times higher among the poorest than among the richest women (-17.7% and -8.9% respectively). The same phenomenon as in Milan was observed in both genders in Turin and Rome, where hospitalisation rates decreased twice as much in the lowest class.

### Inequalities in health care services by period, in relative and absolute terms

Because of the evidence of a statistically significant interaction between income level, time, gender and city, we produced Poisson model estimates for each stratum (Table 2).

**Table 2:** Poisson estimation of relative risk per year and centre

**ROME**													
	**1997**			**1998**			**1999**			**2000**			**Not overlapping 95% CI, 1997 vs 2000**
**Income index**	**RR**	**CI 95%**		**RR**	**CI 95%**		**RR**	**CI 95%**		**RR**	**CI 95%**		

***Male***													
**Q5**	1.00			1.00			1.00			1.00			
**Q4**	1.14	*1.12*	*1.16*	1.12	*1.10*	*1.14*	1.13	*1.11*	*1.15*	1.10	*1.08*	*1.12*	*
**Q3**	1.23	*1.21*	*1.25*	1.21	*1.19*	*1.23*	1.20	*1.18*	*1.22*	1.19	*1.17*	*1.21*	*
**Q2**	1.31	*1.29*	*1.33*	1.31	*1.29*	*1.33*	1.28	*1.26*	*1.30*	1.26	*1.24*	*1.28*	*
**Q1**	1.53	*1.50*	*1.55*	1.50	*1.48*	*1.52*	1.51	*1.48*	*1.53*	1.47	*1.45*	*1.49*	*
***Female***													
**Q5**	1.00			1.00			1.00			1.00			
**Q4**	1.14	*1.12*	*1.15*	1.12	*1.11*	*1.14*	1.13	*1.11*	*1.15*	1.10	*1.08*	*1.12*	*
**Q3**	1.22	*1.20*	*1.24*	1.20	*1.18*	*1.21*	1.19	*1.17*	*1.20*	1.15	*1.14*	*1.17*	*
**Q2**	1.33	*1.31*	*1.35*	1.29	*1.27*	*1.31*	1.29	*1.27*	*1.31*	1.27	*1.26*	*1.29*	*
**Q1**	1.51	*1.49*	*1.53*	1.48	*1.46*	*1.50*	1.47	*1.45*	*1.49*	1.45	*1.43*	*1.47*	*
													
**BOLOGNA**													
													
	**1997**			**1998**			**1999**			**2000**			**Not overlapping 95% CI, 1997 vs 2000**

**Income index**	**RR**	**CI 95%**		**RR**	**CI 95%**		**RR**	**CI 95%**		**RR**	**CI 95%**		

**Q5**	1.00			1.00			1.00			1.00			
**Q4**	1.07	*1.03*	*1.11*	1.05	*1.01*	*1.09*	1.08	*1.04*	*1.13*	1.07	*1.03*	*1.12*	
**Q3**	1.20	*1.16*	*1.24*	1.19	*1.14*	*1.23*	1.13	*1.09*	*1.18*	1.13	*1.08*	*1.17*	*
**Q2**	1.21	*1.17*	*1.26*	1.18	*1.14*	*1.22*	1.19	*1.14*	*1.23*	1.14	*1.09*	*1.18*	*
**Q1**	1.33	*1.29*	*1.38*	1.33	*1.29*	*1.38*	1.31	*1.26*	*1.36*	1.33	*1.28*	*1.38*	
***Female***													
**Q5**	1.00			1.00			1.00			1.00			
**Q4**	1.05	*1.02*	*1.09*	1.07	*1.03*	*1.11*	1.00	*0.97*	*1.04*	1.01	*0.97*	*1.04*	
**Q3**	1.07	*1.03*	*1.10*	1.09	*1.05*	*1.12*	1.08	*1.04*	*1.12*	1.08	*1.04*	*1.12*	
**Q2**	1.14	*1.10*	*1.17*	1.17	*1.13*	*1.21*	1.14	*1.10*	*1.18*	1.12	*1.08*	*1.16*	
**Q1**	1.21	*1.17*	*1.24*	1.23	*1.19*	*1.27*	1.18	*1.14*	*1.22*	1.15	*1.11*	*1.18*	*
													
**MILAN**													
													
	**1997**			**1998**			**1999**			**2000**			**Not overlapping 95% CI, 1997 vs 2000**

**Income index**	**RR**	**CI 95%**		**RR**	**CI 95%**		**RR**	**CI 95%**		**RR**	**CI 95%**		

***Male***													
**Q5**	1.00			1.00			1.00			1.00			
**Q4**	1.09	*1.07*	*1.12*	1.09	*1.06*	*1.11*	1.10	*1.08*	*1.13*	1.10	*1.07*	*1.13*	
**Q3**	1.13	*1.10*	*1.16*	1.14	*1.12*	*1.17*	1.14	*1.11*	*1.16*	1.10	*1.07*	*1.13*	
**Q2**	1.25	*1.23*	*1.28*	1.23	*1.20*	*1.26*	1.25	*1.22*	*1.28*	1.20	*1.17*	*1.23*	*
**Q1**	1.42	*1.39*	*1.46*	1.43	*1.40*	*1.46*	1.49	*1.46*	*1.52*	1.40	*1.37*	*1.44*	
***Female***													
**Q5**	1.00			1.00			1.00			1.00			
**Q4**	1.06	*1.04*	*1.09*	1.04	*1.02*	*1.06*	1.07	*1.05*	*1.10*	1.01	*0.99*	*1.04*	*
**Q3**	1.11	*1.09*	*1.14*	1.13	*1.10*	*1.15*	1.13	*1.11*	*1.16*	1.05	*1.03*	*1.07*	*
**Q2**	1.19	*1.17*	*1.22*	1.18	*1.15*	*1.20*	1.21	*1.18*	*1.23*	1.12	*1.09*	*1.14*	*
**Q1**	1.31	*1.29*	*1.34*	1.29	*1.27*	*1.32*	1.31	*1.28*	*1.34*	1.20	*1.18*	*1.23*	*
													
**TURIN**													
													
	**1997**			**1998**			**1999**			**2000**			**Not overlapping 95% CI, 1997 vs 2000**

**Income index**	**RR**	**CI 95%**		**RR**	**CI 95%**		**RR**	**CI 95%**		**RR**	**CI 95%**		

***Male***													
**Q5**	1.00			1.00			1.00			1.00			
**Q4**	1.19	*1.16*	*1.23*	1.16	*1.13*	*1.20*	1.17	*1.14*	*1.20*	1.12	*1.08*	*1.15*	*
**Q3**	1.24	*1.20*	*1.27*	1.22	*1.18*	*1.25*	1.24	*1.21*	*1.28*	1.20	*1.16*	*1.23*	
**Q2**	1.33	*1.30*	*1.37*	1.32	*1.29*	*1.36*	1.33	*1.30*	*1.37*	1.26	*1.22*	*1.30*	*
**Q1**	1.48	*1.44*	*1.53*	1.44	*1.40*	*1.48*	1.44	*1.40*	*1.48*	1.41	*1.37*	*1.45*	*
***Female***													
**Q5**	1.00			1.00			1.00			1.00			
**Q4**	1.22	*1.19*	*1.25*	1.17	*1.14*	*1.20*	1.17	*1.14*	*1.20*	1.15	*1.12*	*1.19*	*
**Q3**	1.29	*1.26*	*1.32*	1.24	*1.21*	*1.28*	1.26	*1.23*	*1.30*	1.28	*1.25*	*1.32*	
**Q2**	1.37	*1.33*	*1.40*	1.32	*1.29*	*1.35*	1.31	*1.28*	*1.34*	1.30	*1.27*	*1.34*	*
**Q1**	1.48	*1.44*	*1.52*	1.44	*1.40*	*1.47*	1.38	*1.34*	*1.41*	1.41	*1.38*	*1.45*	*

§the model included income level, year, city, gender and age, and is fitted including the year*income*city interaction

Relative risks increased regularly with decreasing income index in Rome and Turin, regardless of gender. Disparities are lower in Bologna than in all other cities in each of the years considered, and often not statistically significant.

### Temporal trend

A decreasing temporal trend of relative risks has been observed in Rome and Turin; a temporal trend was not so clear in Milan, where the gap increased until 1999, while in 2000 it decreased to values below those in 1997 (Table 2), and apparently is not present in Bologna.

## Discussion

Our findings suggest the overall inverse association of CBMI with rates of acute ordinary hospital discharges in all the Italian cities we took into account. This association seems to be coherent with other studies analysing differences in hospitalizations by different SEP indicators. [13,30-34] This inverse association might be interpreted both as the consequence of income-related differences in morbidity, determining a higher need of healthcare among the poorer, and as an unequal utilization of services different income groups. Unfortunately in this study we are not able to disentangle these two possible causes, because we can't adjust neither for health needs or propensity to health services utilizations. However some authors [30,31] have shown that the higher rates of hospitalizations observed among the poorer do not depend only on different health needs but also on a more inappropriate use of services among the less well-off: they showed an equal access to treatments of non-discretionary efficacy but a higher risk of unnecessary treatments.

Other studies conclude that, even taking into account health needs, the economically disadvantaged result to receive a higher number of medical visits and hospitalizations [13].

On the other side some studies have shown a higher use of health services among the more well-off when adjusting for health needs; however these studies uses the number of visits to general practitioners or practising specialists as outcomes instead of hospitalizations and are mainly based on individual income information [35,36].

Our study also showed the deepest relative differentials in hospitalisation rates, between lowest and highest income levels, in Rome and Turin, both for males and females. The narrowest relative differentials were found in Bologna. The observed different absolute values of hospitalisation rates by income level between cities suggest the existence of different explanations of the relative disparities observed. In particular, the low level of inequality in hospitalisations observed in Bologna seems to be related to the higher rates of hospitalisation in the more well-off classes than in other cities.

Turin showed hospitalisation rate ratios similar to those from Rome in all income levels except the lowest, in which rate ratios were slightly lower than those from Rome in each of the years considered. If we assume that income inversely affects hospitalisation on a continuous scale, this may be attributable to the differences in the composition of income classes in the two cities; in fact, absolute values of income are always lower in Rome than in Turin; but the greatest differences correspond to the lowest income levels. The median values in Rome of the first and second deciles were 8,651€ and 10,071€ respectively, and in Turin, 10,329€ and 11,519€. In addition, the income distribution is more asymmetric, with a left tail, and has a higher variability in the first and second decile in Rome than Turin (Rome first decile: mean: 8,166€, sd: 1,650€, first percentile: 475€. Turin first decile: mean: 9,958€, sd: 1,188€, first percentile: 5233€). Hence, even though relative income differences in hospitalisation rates observed in Rome and Turin are similar in dimension, pattern of these differences might be diverse. In fact, in Turin they may be attributable to very low levels of hospitalisation in the richest census tracts, while in Rome to very high levels of hospitalisation among the poorest.

To better understand our result besides the different distribution of income in the four cities we should also consider the general different use of hospitalization in Italian regions. The distribution of standardized hospitalization rates by region published by the Ministry of Health for the year 2000, showing rates ranging from 128 to 208 per 1000 inhabitants, put in evidence how hospitalization can't be considered in our country only as a proxy of health needs, but also reflects different uses of the health care system among regions. These differences also emerge from our results.

We finally observed a generally decreasing trend of hospitalisation rates in all cities in the studied period. This decrease was expected in part, mainly as a consequence of the progressive improvement of forms of health care alternative to hospitalisation. Furthermore, in some of the considered regions, a new system of payment for providers of the NHS was introduced, in 1999 [37], that fixed a maximum number of refundable services on the basis of the expected needs. In fact, this decrease presents different features in the analysed cities and across SEP groups.

We conclude that the decrease in the hospitalisation rate ratio between the lowest and highest level of income in Rome, Turin and Milan (only for females) is mainly due to the greater decrease of hospitalisations among the poorest than among the richest. Assuming a stable demographic composition within SEP groups in the city populations analysed, this may indicate a trend towards a generally more appropriate use of hospitalisation.

The different scenarios of decline of hospitalization rates observed suggest, besides eventual differences in population health, the underlining effect of different regional health policies concerning hospitalisation care, their effect over time and population groups, and the regional heterogeneity of absolute income impact on hospitalisation, in addition to that produced by relative income position. In fact, Italy is characterized by a National Health System, that is differently planned, administered and organized at the regional level; hence, local results on inequalities in health services utilization can be interpretable with specific reference to the regional context, and data extension at the national level could be desirable, as well as feasible.

Different limitations are present in our study. The validity of income as an indicator of socio-economic status obviously depends on the quality of tax registry data. Tax evasion could produce an underestimate overall wellness with a major bias among the richest. In this case, the use of an indicator for small geographic areas instead of individuals could in part limit the risk of this bias. The limitations and advantages of geographical and individual indicators have been widely studied; the use of a small area indicators can produce misclassification, which tends, if not differential, to produce an underestimation of the studied association. However, studies of the association between hospitalisation and socio-economic status using small area and individual indicators have produced similar results [25]. Some authors have demonstrated that inferring individual from CB level SEP reduces estimates of risk ratios, and therefore much larger numbers of observations would be needed to better understand the relationship of SEP to survival, and other disease outcomes [38,39]. This also could be true for our study. But the very large population we considered should have protected from underestimating the risk.

Furthermore, as already stated in the introduction, we have to underline the general difficulties in interpreting the association between income and hospitalization, as this is affected by many concomitant conditions as population health needs pattern, regional health policies, level of utilization of heath services, appropriateness and equity in the utilization of services. Some clues on the reciprocal weight of all these components in the explanation of inequalities in hospitalisation will be obtained through the analysis of hospitalisation for specific health conditions [40,41]. Nevertheless this was a pilot work, aimed at understanding the performance of this indicator in the more general situation, before to implement more specific studies.

Finally we excluded hospitalizations of residents in other regions. Because this phenomenon presumably affects the more well-off more than the poorer, it might induce an underestimate of hospitalization rates in the higher income classes. However because we have only considered large cities (with the only possible exception of Bologna), this should be a limited phenomenon.

In conclusion our paper proposes a methodology for the construction of a small area economic indicator, based on total population fiscal data, available and comparable at the National level, that can be potentially updated every year. Under this point of view, our results contribute to the improvement of tools for monitoring inequalities in health and in health services utilization, besides suggesting hypotheses for further investigations of temporal and regional trends.

## Competing interests

The authors declare that they have no competing interests.

## Authors' contributions

PS conceived of the study, participated to its design coordination and analysis, CM participated to the study design and analysis, GC participated in designing the database and analysis design, VB made the data management and participated to the analysis, NC participated to the study design and data analysis, AR participated to the study design and data analysis, CAP conceived of the study and participated to its design. All authors read and approved the final manuscript.
